# Traditional Medicinal Herbs and Food Plants Have the Potential to Inhibit Key Carbohydrate Hydrolyzing Enzymes *In Vitro* and Reduce Postprandial Blood Glucose Peaks *In Vivo*

**DOI:** 10.1100/2012/285284

**Published:** 2012-02-20

**Authors:** M. Fawzi Mahomoodally, A. Hussein Subratty, A. Gurib-Fakim, M. Iqbal Choudhary, S. Nahar Khan

**Affiliations:** ^1^Department of Health Sciences, Faculty of Science, University of Mauritius, Reduit 230, Mauritius; ^2^Centre for Phytotherapy Research, Cybercity 2, 7th Floor, Ebene 230, Mauritius; ^3^H. E. J. Research Institute of Chemistry and International Center for Chemical and Biological Sciences, University of Karachi, Karachi 75270, Pakistan; ^4^Department of Pharmacy, East West University, 43 Mohakali C/A, Dhaka 1212, Bangladesh; ^5^Department of Molecular and Cellular Biology, Harvard University, 52 Oxford Street, MA 02138, USA

## Abstract

We hypothesized that some medicinal herbs and food plants commonly used in the management of diabetes can reduce glucose peaks by inhibiting key carbohydrate hydrolyzing enzymes. To this effect, extracts of *Antidesma madagascariense* (AM), *Erythroxylum macrocarpum* (EM), *Pittosporum senacia* (PS), and *Faujasiopsis flexuosa* (FF), *Momordica charantia* (MC), and *Ocimum tenuiflorum* (OT) were evaluated for *α*-amylase and *α*-glucosidase inhibitory effects based on starch-iodine colour changes and PNP-G as substrate, respectively. Only FF and AM extracts/fractions were found to inhibit *α*-amylase activity significantly (*P* < 0.05) and coparable to the drug acarbose. Amylase bioassay on isolated mouse plasma confirmed the inhibitory potential of AM and FF extracts with the ethyl acetate fraction of FF being more potent (*P* < 0.05) than acarbose. Extracts/fractions of AM and MC were found to inhibit significantly (*P* < 0.05) *α*-glucosidase activity, with IC_50_ comparable to the drug 1-deoxynojirimycin. *In vivo* studies on glycogen-loaded mice showed significant (*P* < 0.05) depressive effect on elevation of postprandial blood glucose following ingestion of AM and MC extracts. Our findings tend to provide a possible explanation for the hypoglycemic action of MC fruits and AM leaf extracts as alternative nutritional therapy in the management of diabetes.

## 1. Introduction

Public interest in complementary and alternative therapies, including the use of botanical and natural dietary supplements has witnessed spectacular rise throughout the world. Indeed, knowledge of the therapeutic and nutritional properties of medicinal herbs and food plants predates recorded history [1–5]. The use of and search for drugs and botanical supplements derived from plants have accelerated in recent years [6–8]. Ethnopharmacologists, botanists, microbiologists, nutritionists, and natural-products chemists are combing the earth for phytochemicals and “leads” which could be developed for the treatment of various ailments [8–10]. Evidence of the beneficial therapeutic effects of these medicinal herbs is seen in their continued use [5, 6, 11]. One pathology where medicinal plants have been extensively used is diabetes mellitus (DM) [10, 12, 13]. This condition is the world's largest endocrine disease involving metabolic disorders of carbohydrate metabolism characterized by fasting elevation of blood glucose level [14]. While the cause of elevated blood glucose may be associated with either too little or too much insulin, the complications of chronically high serum glucose are devastating to the individual. If untreated, DM can lead to severe complications. Patients with diabetes experience significant morbidity and mortality from microvascular and macrovascular complications [15].

DM is becoming a devastating scourge and despite the recent surge in new drugs to treat and prevent the condition, its prevalence continues to soar. To this effect, nutritional therapies including the use of alternative traditional medicinal plant systems and herbal food with various principles and properties have witnessed renewed interest in the last few decades [5–8, 16]. Indeed, along with dietary measures, plant preparations formed the basis of the management of this disease even after the introduction of insulin [11]. The beneficial multiple activities like manipulating carbohydrate metabolism by various mechanisms, preventing and restoring integrity and function of *β*-cells, insulin-releasing activity, improving glucose uptake and utilization by medicinal plants and inhibition of digestive enzymes offer exciting opportunity to develop them into novel nutritional therapeutics [17].

Recently, there have been a growing number of publications on the potential of antidiabetic medicinal herbs and food plants to inhibit *α*-amylase and *α*-glucosidase. Indeed, advances in understanding of the activity of *α*-amylase and *α*-glucosidase have led to the development of new pharmacologic agents [18]. It is believed that *α*-amylase inhibition has gastrointestinal and metabolic effects that may aid not only in the treatment of postprandial hyperglycemia but also of obesity. Weight reduction diets and weight loss schemes have enjoyed a unique popularity in many parts of the world. Recently, the production and marketing of *α*-amylase and *α*-glucosidase inhibitors to block the digestion and absorption of ingested carbohydrate (mainly starch) has been promoted as a quick weight loss scheme. Claims of a “remarkable new natural food supplement: termed as carbohydrate or starch blockers” suggest that dieters may eat carbohydrate-rich foods without experiencing the weight gain of increased caloric consumption [2, 18, 19].

Currently, many indigenous and exotic herbs and food plant species of Mauritius and other Mascarene Islands have been used in folkloric medicine to treat various ailments of man including chronic diseases such as DM [5–8]. Several kinds of extracts from various exotic and indigenous herbs and food plants are sold as decoctions or “tisanes” in several markets to treat minor ailments and commonly used as nutritional supplements. Nonetheless, even with this vast array of data, few medicinal herbs and food plants of Mauritius have been scientifically evaluated for their possible medicinal application [20, 21].

In the present study medicinal herbs and food plants of Mauritius which are traditionally and routinely used as dietary adjuncts for the management of diabetes were evaluated for their blood glucose lowering potential *in vitro* and *in vivo*. Our first objective in this endeavor comprised of an initial evaluation of the crude extracts and respective fractions for any *α*-amylase and *α*-glucosidase inhibitory potential *in vitro*. Our second objective was to corroborate any inhibitory potential using the isolated mouse plasma assay *in vitro*. Finally, only potent crude extracts showing significant inhibition *in vitro* were further evaluated for any possibility to decrease postprandial glucose in glycogen-loaded mice *in vivo*. It is expected that results from this research might support the rationality of these herbs and food plants as potential dietary adjuncts in the management of diabetes.

## 2. Materials and Methods

### 2.1. Plant Materials

Many of the plants chosen in this study are used by the Mauritian population not only for food purposes but also form a part of the local pharmacopoeia for the management of diabetes. Briefly, leaves of *Antidesma madagascariense *(Euphorbiaceae), *Erythroxylum macrocarpum *(Erythroxylaceae),* Pittosporum senacia *(Pittosporaceae), and* Faujasiopsis flexuosa* (Asteraceae) were collected from Petrin and Forest-Side, Mauritius. The Curator of the National Herbarium confirmed the identity of the plants. Holy basil (*Ocimum tenuiflorum*—Lamiaceae) was obtained from the University of Mauritius' farm. Fresh unripe fruits of bitter melon, *Momordica charantia *(Cucurbitaceae) were obtained from the local market of the island and prepared as described previously [4–8]. The plant materials were oven-dried for several hours or air-dried in a drying cabinet for 4 to 5 days until constant mass was obtained. The dried plant materials were then homogenized in an electrical food grinder to a fine powder and stored in well-sealed plastic containers [4–8].

### 2.2. Extraction and Fractionation

Powdered (10 g) plant materials were extracted to exhaustion with 50 mL of water in a Soxhlet apparatus for 5 hours as described previously [4–8]. The solvent was then distilled off under reduced pressure and temperature (40°C) to afford crude extracts. The extracts were concentrated in vacuo using a rotary evaporator (Model Buchi rotavapor R-114, Switzerland). The resultant concentrate was measured and the gummy material collected in the appropriate solvent for examination. The paste-like suspension was diluted in the extraction solvent (water) for further experiments.

Methanolic extracts were obtained by triple soaking in 80% methanol at room temperature for 3 days [22]. Crude methanolic and aqueous extracts were obtained by removing the solvent under reduced pressure. The extracts were concentrated *in vacuo *using a rotary evaporator. The paste-like suspension was diluted in DMSO for further experiments. Crude methanolic extracts were fractionated by solvent-solvent extraction procedure into dichloromethane, ethyl-acetate, *n*-butanol, and aqueous fractions for two successive 24 hr periods respectively [22]. In all, 6 different extracts/fractions for each plant were tested for amylase and glucosidase assays *in vitro*.

### 2.3. Amylase Inhibitory Assays

The activity of *α*-amylase was carried out according to the starch-iodine colour changes, [23] with minor modifications as we described previously [24]. 0.1 mL of *α*-amylase solution (15 *μ*g/mL in 0.1 M Acetate buffer, pH 7.2 containing 0.0032 M Sodium Chloride) was added to a mixture of 3 mL of 1% soluble starch solution (1 g soluble potato starch, suspended in 10 mL water was boiled for exactly 2 minutes. After cooling, water was added to a final volume of 100 mL. The solution was kept in the refrigerator and was used within 2-3 days) and 2 mL acetate buffer (0.1 M, pH 7.2) preequilibrated at 30°C in a water bath. Substrate and *α*-amylase blank determinations were undertaken under the same conditions. At zero time and at the end of the incubation period, 0.1 mL of reaction mixture was withdrawn from each tube after mixing and discharged into 10 mL of an Iodine solution (0.245 g Iodine and 4.0 g Potassium Iodide in 1 liter). After mixing, the absorbancy of the starch-iodine mixture was measured immediately at 23°C at 565 nm using a spectrophotometer. The absorbancy of the starch blank was subtracted from the sample reading. One unit of amylase activity was arbitrarily defined as

(1) $$\left[\frac{A_{o}-A_{t}}{A_{o}}\right]\times 100,$$

where *A*_*o*_ and *A*_*t*_ were absorbances of the iodine complex of the starch digest at zero time and after 60 min of hydrolysis. Specific activity of amylase was defined as units/mg protein/60 min. Under experimental conditions the starch blank did not change after 24 hours. For longer incubation periods, the absorbancy of the enzyme blank was used to correct experimental values [23, 24]. 0.10 mL of the aqueous plant extract solution was incubated with 0.1 mL of the enzyme and substrate solution for 15 minutes at 30°C. The assay was conducted as described above; one unit of amylase inhibitor was defined as that which reduced the activity of the enzyme by one unit. The different extracts and fractions were diluted as appropriate to establish any dose-dependent effects and for calculation IC_50_ values. Assays were replicated three times throughout the study and the mean values used [23, 24].

### 2.4. Amylase Assay in Mouse Plasma

Five- to 6-weeks-old male mice maintained on commercial feed and tap water *ad libitum* were used for this study. They were housed in standard environmental conditions with 12 hr light and 12 hr dark exposure. Investigations using experimental animals were conducted in accordance with internationally accepted principles for laboratory animal use and care. After overnight fasting, mice were killed by a severe blow on the head against a hard surface with subsequent cervical dislocation. Cardiac blood was collected with a heparin-treated cylinder and centrifuged at 5000 g for 20 minutes. The plasma was collected and stored at 4°C until further use [25, 26].

Amylase activity was carried out according to the Iodo-Starch method [25, 26]. 0.5 mL of substrate buffer solution (0.25 M/L phosphate buffer at pH 7.0 containing 40 mg/dL soluble starch) was mixed well with 100 *μ*l of distilled water and kept at 37°C for 5 minutes. This was done for both the sample and blank. After the incubation period, 0.01 mL of mouse plasma was added to the sample solution only and well mixed before reincubated at 37°C for 7.5 minutes. After the second incubation, 0.5 mL of coloring reagent (0.254 g Iodine and 4.0 g Potassium Iodide in 1 L) and 2.5 mL of distilled water were added. After mixing, the absorbance of the solution was measured at 650 nm using a spectrophotometer (Cecil 1020) at 520 nm.

0.10 mL of the serially diluted aqueous plant extract was incubated with 0.5 mL of substrate buffer solution at 37°C for 5 minutes. The same procedure was adopted as above. Triplicate assays were performed throughout the study and mean values were used [25, 26]. The amylase unit was calculated by using the following equation:

(2) $$\left[\frac{E_{B1}-E_{S}}{E_{B1}}\right]\times 800,$$

where *E*_*S*_ is the absorbance of the sample solution and *E*_*B*1_ is the absorbance of the blank solution.

The percentage inhibitory activity was calculated using the following formula: (*A* − *B*/*B*) × 100, where *A* is the activity of the enzyme with test solution and *B* is the activity of the enzyme without test solution.

### 2.5. *In Vitro* *α*-Glucosidase Assays

*α*-Glucosidase enzyme inhibition assay was performed as described previously [27–29] with minor modifications [30]. *α*-Glucosidase was from Wako Pure Chemical Industries Ltd (Wako 076-02841). The inhibition was measured spectrophotometrically in the presence of the extracts/fractions or positive control at pH 6.9 and at 37°C using 0.7 mM *p*-nitrophenyl *α*-D-glucopyranoside (PNP-G) as a substrate and 250 units/mL enzyme, in 50 mM sodium phosphate buffer containing 100 mM NaCI. The rate of release of *p*-nitrophenol from PNP-G in the presence of each extract/fraction was quantified. 1-Deoxynojirimycin (Sigma) was used as positive control. The increment in absorption at 400 nm due to the hydrolysis of PNP-G by *α*-glucosidase was monitored continuously with the spectrophotometer equipped with an ELISA microtitre plate (Molecular Devices USA). The IC_50_ value was defined as the concentration of *α*-glucosidase inhibitor to inhibit 50% of its activity under the assay conditions [30].

### 2.6. *In Vivo* Studies

 Seven mice (5- to 6 week-old male) were used for each test group as described previously [25, 26]. Each were kept in individual cages, maintained under 12/12 h light/dark cycles at room temperature (22–25°C). They were fed commercial stock diet and water. Mice were deprived of food for 16 h before experimentation, but allowed free access to tap water throughout the experiment. The positive control used was acarbose at a dosage of 400 mg/kg mouse [25, 26]. Glucose concentration was determined using the glucose oxidase kit [4]. Glycogen, acarbose, and extracts were administered orally by intragastric route. Blood samples were obtained by the tail venipuncture method after 60 minutes after oral administration of the extracts.

### 2.7. Phytochemical Screening

Fruits, leaves, and twigs where appropriate were subjected to a thorough phytochemicals screening using standard protocols [5, 6, 8] to detect the presence of the following secondary metabolites: alkaloids, coumarins, terpenes, anthraquinones, tannins, phenols, leucoanthocyanins, flavones, and saponins.

### 2.8. Statistical Analysis

All data are expressed as mean ± SEM for three experiments except otherwise stated. The difference between the mean ± SEM of the *α*-amylase activity (units/mg protein/60 min) and *α*-glucosidase between the control (without extract) and experimental group were assessed using the One Way Analysis of Variance (ANOVA) test. *P* values less than 0.05 (*P* < 0.05) were considered statistically significant [31]. Percentage inhibitions compared to the control experiments were calculated as describe previously [32]. The IC_50_ value was defined as the concentration of *α*-amylase/glucosidase inhibitor to inhibit 50% of its activity under the assay conditions [30]. Data manipulation and statistical analyses were performed using Excel software (Microsoft 2007) and SPSS version 16.0 for Windows 7.

## 3. Results

### 3.1. Amylase Activity *In Vitro*

Data obtained on the effect of the medicinal plants showed that the different plant extracts exhibited variable inhibitory effects on *α*-amylase activity *in vitro*. The results are presented in Tables 1 and 2. In this study, only the two plants: AM and FF were found to inhibit amylase activity significantly (*P* < 0.05) and in a dose-dependent manner. The concentrations of the extracts and organic fractions tested ranged from 62.5 to 2000 *μ*g/mL.

However, it is clear from the results that the two different plants extracts (AM and FF) exhibit considerable differences in degree of inhibition for all the concentrations tested. For instance, the highest percentage inhibitory activity of AM was 95.26% at a concentration of 2 mg/mL compared to FF (−87.54 ± 12.37 at a similar concentration). Extracts of MC fruits, EM, and OT leaves did not have any significant inhibitory effects (*P* > 0.05) on *α*-amylase activity with increasing graded concentrations (data not included). At higher concentration (2000 *μ*g/mL), MC leaf extract was found to posses significant (*P* < 0.05) inhibitory effects. A similar significant (*P* < 0.05) inhibitory effect was observed for the ethyl acetate fraction of EM at 2000 *μ*g/mL (results not shown). On the other hand, FF significantly (*P* < 0.05) inhibited *α*-amylase activity at lower doses (56.14 ± 1.16 at 62.5 *μ*g/mL) compared to AM 46.23 ± 5.63 at 62.5 *μ*g/mL. Hence it can be suggested that the concentration of the inhibitory phytochemical(s) in FF leaf extracts was higher than in AM.

**Figure 1:**
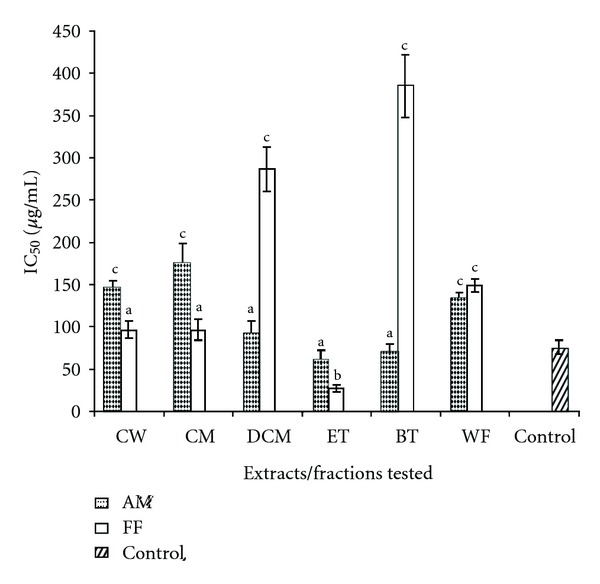
IC_50_ values (*μ*g/mL) for the active extracts/fractions of AM and FF against *α*-amylase activity. CW: crude water; CM: crude methanol; DCM: dichloromethane; ET: ethylacetate; BT: *n*-butanol; WF: water fraction. Positive control: Acarbose. ^a^Values comparable to positive control. ^b^Values significantly lower (*P* < 0.05) from positive control. ^c^Values significantly higher (*P* < 0.05) compared to positive control.

Figure 1 summarizes the IC_50_ values (*μ*g/mL) for the active extracts and fractions of AM and FF against *α*-amylase activity. Only AM and FF were selected to compute the IC_50_ values as they were the most potent extract that gave significant (*P* < 0.05) *α*-amylase inhibitory effects. The IC_50_ ranged from 61.52 ± 11.09 *μ*g/mL to 175.97 ± 22.96 *μ*g/mL for AM and 27.36 ± 4.17 *μ*g/mL to 384.72 ± 39.9 *μ*g/mL for FF. In both cases, it was the ethylacetate fraction that gave the best inhibitory activities (61.52 ± 11.09 *μ*g/mL for AM and 27.36 ± 4.17 *μ*g/mL for FF). For AM the highest IC_50_ values were recorded for the crude water and methanol extracts (145.21 ± 9.36 and 175.97 ± 22.96 *μ*g/mL, resp.), whereas for FF the highest IC_50_ values were recorded for *n*-butanol and dichloromethane fractions (384.72 ± 39.97 *μ*g/mL and 286.70 ± 26.67 *μ*g/mL, resp.).

It was noted that the water extract, methanol, and ethyl acetate fractions of FF gave similar values as the standard antiamylase drug acarbose, whereas dichloromethane, ethyl acetate and *n*-butanol fractions of AM showed lower activity than acarbose (IC_50_ value of 75.86 ± 8.16). It is noteworthy to highlight that ethylacetate fraction of FF gave IC_50_ value significantly lower than the antiamylase drug acarbose.

### 3.2. Amylase Activity in Mouse Plasma

To further substantiate and for comparative purpose, the inhibitory properties of the most active fractions of AM and FF were further investigated for possible inhibition on amylase activity in mouse plasma. In this study the ethyl acetate fraction of both plants were tested in the mouse plasma as it was this fraction that resulted in greatest significant (*P* < 0.05) and dose-dependent amylase inhibition at all concentrations tested.

**Figure 2:**
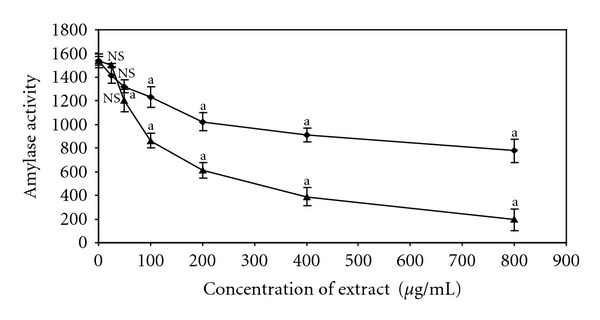
Graded concentrations of ethylacetate fractions of AM and FF on amylase activity in mouse plasma. The results are expressed as mean ± S.E.M of seven observations in each group. Amylase unit expressed as described by Kobayashi et al. [25, 26] in mouse plasma. AM (circles) and FF (triangles). ^a^ *P* < 0.05 compared to control. NS, not statistically significant compared to control (without extract).

The amylase activity in mouse plasma is summarized in Figure 2. AM was found to inhibit amylase activity significantly (*P* < 0.05) from 7.80 to 49.37% whilst FF inhibit amylase activity significantly from 7.54 to 87.39% with increasing graded concentrations of the extracts (from 100 and 800 *μ*g/mL) in mouse plasma. AM was found to inhibit amylase significantly (*P* < 0.05) at 100 *μ*g/mL unlike FF which was efficient at lower concentration of 50 *μ*g/mL.

In addition, the inhibitory effect of FF in mouse plasma in the present study appears to be concentration dependent. Although there was no significant (*P* > 0.05) effect at lower concentration (25 *μ*g/mL), the higher concentrations (above 100 *μ*g/mL) did lead to significantly (*P* < 0.05) diminished amylase activity. This inhibition in mouse plasma further supports the reduction of amylase activity presented in Table 1. Results from this *in vitro* assay also tend to show that higher dose (100 *μ*g/mL) of FF and AM was needed to produce significant inhibition (*P* < 0.05) in the mouse plasma unlike the previous experiments (50 *μ*g/mL) as depicted in Table 1. This difference might be attributed to the concentration of amylase in the mouse plasma.

### 3.3. *α*-Glucosidase Activity *In Vitro*

*α*-Glucosidase activity was assessed by measuring the rate of release of *p*-nitrophenol from *p*-nitrophenyl *α*-D-glucopyranoside.  Table 2 summarizes the results obtained. It should be noted that unlike *α*-amylase assay, here the *α*-glucosidase activity was carried in microtiter plates and hence the concentrations of extracts tested ranged from 100 to 6.25 *μ*g/mL. Here, the range of concentrations of the extracts tested was comparatively smaller as compared to the *α*-amylase assays which might be due to the concentration of the substrate and enzymes in each assay.

**Figure 3:**
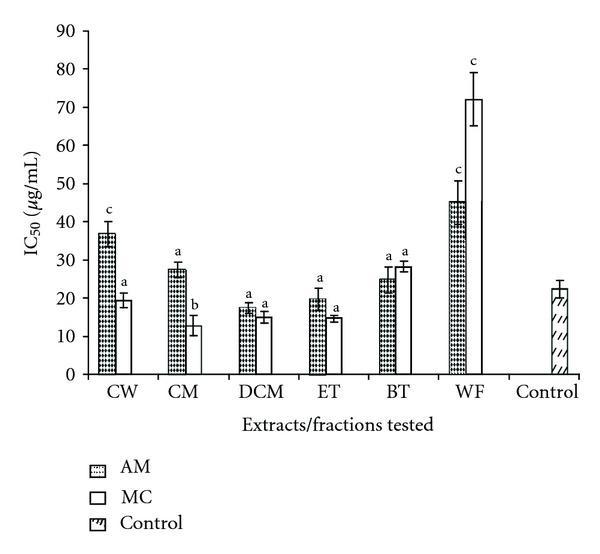
Summary of IC_50_ values (*μ*g/mL) for the active extracts/fractions of AM and MC against *α*-glucosidase activity. CW: crude water; CM: crude methanol; DCM: dichloromethane; ET: ethylacetate; BT: *n*-butanol; WF: water fraction. Positive control: 1-Deoxynojirimycin. ^a^Values comparable to positive control (1-Deoxynojirimycin). ^b^Values significantly lower (*P* < 0.05) from positive control. ^c^Values significantly higher (*P* < 0.05) compared to positive control.

As compared to *α*-amylase activity, here also the best activity was observed for AM and also for MC extracts. The percentage inhibition ranged from 14.32 ± 1.62 to 98.64 ± 7.42 and 26.63 ± 2.07 to 90.56 ± 8.54 for AM and MC, respectively. Slight inhibitory activity was also recorded for FF extracts; however this effect was not observed for all the concentrations tested (data not included). To this effect, IC_50_ values of only AM and MC were calculated and compared to the standard drug 1-Deoxynojirimycin as depicted in Figure 3.

The IC_50_ values ranged from 19.70 ± 2.87 *μ*g/mL to 44.92 ± 5.67 *μ*g/mL and 12.72 ± 2.65 *μ*g/mL to 72.12 ± 6.97 *μ*g/mL for AM and MC, respectively, as shown in Figure 3. The lowest IC_50_ value was recorded for ethyl acetate fraction for both plants. Both water and methanol crude extracts and respective fractions of AM and MC were found to inhibit the enzyme *α*-glucosidase similar to the standard drug 1-Deoxynojirimycin (Sigma) except water fraction. However, the crude methanol and dichloromethane fractions were found to have IC_50_ values lower than the standard positive control.

### 3.4. Change in Blood Glucose Level in Glycogen-Loaded Mouse

Extracts (AM, FF, and MC) which showed significant and concentration-dependent inhibition of amylase in isolated mice plasma and *α*-glucosidase *in vitro *were further evaluated *in vivo*. Only the crude water and methanol extracts together with the ethylacetate fraction were selected for investigation *in vivo* as it has showed the best amylase and glucosidase inhibitory activities (Tables 1 and 2). They were all evaluated for possible depressive effect as compared to standard drug acarbose following the rise in blood glucose level in glycogen-loaded mice. The alteration in blood glucose level 60 mins after oral administration of each extract (1 g and 2 g per kg of mice for AM, FF, and MC) and glycogen (2000 mg/kg) is depicted in Table 3. Crude water and methanol extracts of FF were found not to possess significant (*P* < 0.05) inhibitory effects *in vivo* as compared to its *in vitro* amylase inhibitory activities. Only AM and MC extracts/fractions significantly (*P* < 0.05) repressed the increase in blood glucose concentration in mice, comparable to acarbose (positive control tested at 400 mg/kg). The ethylacetate fraction was also found to be more potent fraction and showed glucose-lowering properties (−59.4%) comparable to acarbose (−55.1%).

### 3.5. Phytochemical Analysis

Table 4 shows the results from phytochemical screening of the six medicinal plant extracts. Tannins were present in all the tested extracts, whereas anthraquinones were absent in all tested extracts.

## 4. Discussion 

The present investigation was geared towards evaluating the potential of common medicinal herbs and food plants to inhibit key carbohydrate hydrolyzing enzymes *in vitro* and any possibility to decrease postprandial glucose in glycogen-loaded mice *in vivo*. Nutritionally, carbohydrates are the core components of the human diet which consist mainly of complex sugars such as starch and glycogen (~60%), and disaccharides such as sucrose (~30%). These must be hydrolyzed by gastrointestinal enzymes before they can be transported through the mucosa of the bowel. *α*-Amylase is a key digestive enzyme responsible for hydrolyzing starch to maltose, which further breaks to glucose prior to absorption in the small intestine. A few minutes after the ingestion of carbohydrate-rich food a marked hyperglycemia leading to hyperinsulinemia is observed [33, 34] which are both highly undesirable in patients suffering from NIDDM, obesity or hyperlipoproteinemia. Inhibition of *α*-amylase enzyme should reduce the unfavorable high postprandial blood glucose peaks observed in diabetics; therefore acting as “carbohydrate blockers” [35, 36]. Drugs, which reduce postprandial hyperglycemia by suppressing the hydrolysis of carbohydrates, have been shown to be effective for the prevention and treatment of NIDDM [16]. Endoglucanases, such as *α*-amylase that catalyze hydrolysis of the internal *α*-1,4-glucosidic linkage in starch and other related polysaccharides, have also been the target of medicinal plants investigations as potential candidates for the suppression of postprandial hyperglycemia [18, 34]. On the other hand, *α*-glucosidase is a membrane-bound enzyme, located at the epithelium of the small intestine, which catalyses the cleavage of glucose from disaccharides and oligosaccharides. A review of the scientific literature tends to show that a significant contribution has been made regarding *α*-glucosidase inhibitors and these were the first drug developed to meet the needs of better postprandial glucose control when sulfonylureas and biguanides were the only available oral antidiabetics therapy [37]. Inhibition of *α*-glucosidase and *α*-amylase can significantly decrease the postprandial increase of blood glucose after a mixed carbohydrate diet and therefore can be an important strategy in the management of postprandial blood glucose level in NIDDM patients and borderline patients. Hence the attractive targets like *in vitro *inhibition of *α*-glucosidase and *α*-amylase enzymes are currently in vogue. 

Interesting, results from the present investigation tend to show that extracts of these medicinal herbs and food plants exhibit variable inhibitory effects on *α*-amylase and *α*-glucosidase activity *in vitro*. However, only two medicinal plants (AM and FF) were both found to possess significant anti-*α*-amylase activities. It is obvious from their respective percentage inhibition that the two different plant extracts exhibited considerable different degrees of inhibition and dose-dependency relationship. For instance, the highest percentage inhibitory activity of FF was observed at lower concentration unlike AM. On the other hand, FF was seen to significantly inhibit *α*-amylase activity at low doses compared to AM and hence can be suggested to be more potent. The IC_50_ values also showed that dichloromethane, *n*-butanol, and ethylacetate fractions of AM produced similar inhibitory effects as the standard drug acarbose. The lowest IC_50_ value was obtained from the ethylacetate fraction of FF, which was lower than acarbose. It is obvious that results amassed from this study would tend to suggest that the active phytochemicals are higher in these specific fractions as compared to the crude extracts and hence it can be argued that the concentrations of the inhibitory phytochemical(s) in FF are higher than AM. Results from the *α*-amylase assay in isolated mouse plasma showed that higher dose of FF was needed to produce significant inhibition unlike previous starch-iodo *in vitro* experiment in the present study. This difference might in turn be attributed to the concentration of *α*-amylase in the mouse plasma. 

In relation to the *α*-glucosidase assay, AM and MC extracts were found to possess significant inhibitory effects against *α*-glucosidase activity in a dose-dependent manner. The glucosidase inhibitory effects correlate to some extent with *α*-amylase inhibitory effects observed as AM was found to be active against both enzymes. To further appraise and confirm the observed *in vitro* inhibitory properties of AM and FF extracts, *in vivo* studies were conducted in glycogen-loaded mice. However, it was found that only crude water and methanol extract of MC and AM depicted significant inhibition of postprandial glucose rise and comparable to acarbose activity and the ethylacetate fraction being the most active fraction. 

 In the present study, considerable differences in the inhibitory activities were observed towards these key carbohydrate enzymes which suggest the susceptibility of the *α*-amylase and *α*-glucosidase molecule to various secondary metabolites present in these two plants. It is to be noted that important constituents for the inhibitory activity against *α*-amylase are mainly polyphenolic compounds and the composition of which in turn varies considerably from species to species, climatic conditions, and the physiological state of developments of these medicinal herbs and food plants [38]. Interestingly, the phytochemical analysis indicated the presence of flavonoids and tannins along with other major common secondary metabolites in the leaves of AM and FF extract. Previous reports on *α*-glucosidase inhibitors isolated from medicinal plants suggest that several potential inhibitors belong to flavonoids glycoside class which has the characteristic structural features to inhibit *α*-glucosidase enzyme. MC fruit on the other hand, has been found to possess a wide array of bioactive phytochemicals and several classes of biologically active saponins have been isolated. Bioactive saponins constituents of natural products have been found to suppress the transfer of glucose from the stomach to the small intestine and inhibit glucose and fluid transport at the brush border membrane [4, 8, 39, 40]. Since these classes of phytochemicals are known to have established biological activities, hence their presence might to some extent justify the inhibitory activities on *α*-amylase and *α*-glucosidase observed herein. Additionally, the significant inhibitory properties might also be explained due to the presence of dietary fiber which is known to delay carbohydrate digestion and absorption. To this effect, it is most probable that the synergistic effects of these secondary metabolites together with dietary fiber might be present in sufficiently high amounts to delay absorption of glucose observed from the present *in vivo* study. Our study also tends to confirm previous biological activity observed from MC, where an aqueous methanolic extract of MC seeds was found to possess 1-deoxynojirimycin-like inhibitory properties [40]. However, the present study is the first report of glucosidase inhibition from MC fruits. Additionally, we have evaluated the different crude water extracts of these plants as they are commonly prepared (most commonly used as vegetables) and ingested in aqueous form by the local people. In the traditional pharmacopoeia of Mauritius and also some Asian countries, these plants have been reported to possess blood sugar lowering properties in diabetic patients after taking aqueous decoctions of the plants. Hence these results appear to validate the medicinal claims and also the way people use these herbal remedies as food sources. To this effect, it can be suggested that reduction in amylase and glucosidase activity exhibited by these plants might reduce postmeal blood sugar peaks and hence slowing gastric emptying. If results from the present *in vivo* study could be extended to human, consequently it would be tempting to suggest that ingestion of leaf and fruit decoctions of these medicinal herbs and food plants with carbohydrate-rich foods can be of some help to reduce or delay postprandial hyperglycemia in diabetic patients. Indeed, postprandial hyperglycemia strongly depends on the amount of absorbed monosaccharides and the velocity of absorption in the small intestine. However, it should be noted that a battery of *in vivo *tests as well as randomized controlled clinical studies should be conducted on these plants to confirm whether the *in vitro/in vivo *results reported here translate into activities that might support the traditional uses of these food plants in humans. 

## Figures and Tables

**Table 1:** Effects of AM and FF on *α*-amylase activity.

Concentration of extracts (*μ*g/mL)	*α*-amylase activity % inhibition (−) or stimulation (+)^a^					
	Crude water extract	Crude methanol Extract	Dichloro-methane fraction	Ethyl acetate fraction	*n*-Butanol fraction	Water fraction
62.5	(−28.53 ± 1.31*) [−40.78 ± 3.56*]	(−36.26 ± 3.64*) [−48.29 ± 3.65*]	(−43.22 ± 0.59*) [−33.59 ± 5.61*]	(−46.23 ± 5.63*) [−56.14 ± 1.16*]	(−42.29 ± 1.61*) [−28.39 ± 0.64*]	(−27.03 ± 1. 56*) [−37.98 ± 2.64*]
125	(−52.56 ± 2.15*) [−61.25 ± 2.86*]	(−48.56 ± 2.25*) [−50.16 ± 2.68*]	(−59.36 ± 4.56*) [−42.29 ± 3.34*]	(−62.36 ± 7.62*) [−67.84 ± 3.75*]	(−64.56 ± 6.53*) [−33.14 ± 1.23*]	(−65.23 ± 3.47*) [−47.34 ± 3.21*]
250	(−64.54 ± 3.45*) [−65.29 ± 7.21*]	(−53.12 ± 8.58*) [−58.23 ± 6.89*]	(−60.24 ± 6.42*) [−50.17 ± 2.65*]	(−78.36 ± 5.26*) [−68.59 ± 7.64*]	(−74.31 ± 4.28*) [−38.49 ± 8.76*]	(−72.56 ± 3.58*) [−63.95 ± 9.56*]
500	(−75.34 ± 5.68*) [−71.28 ± 6.41*]	(−63.32 ± 4.54*) [−60.59 ± 11.74*]	(−67.52 ± 5.98*) [−57.23 ± 10.94*]	(−83.31 ± 6.14*) [−74.19 ± 9.34*]	(−79.45 ± 9.36*) [−57.28 ± 1.87*]	(−77.15 ± 4.51*) [−68.34 ± 10.31*]
1000	(−87.54 ± 4.56*) [−72.39 ± 9.61*]	(−70.12 ± 7.87*) [−65.36 ± 8.53*]	(−70.54 ± 8.61*) [−61.86 ± 6.75*]	(−93.12 ± 6.53*) [−83.65 ± 7.86*]	(−87.16 ± 9.86*) [−63.18 ± 9.65*]	(−80.14 ± 7.82*) [−70.64 ± 12.15*]
2000	(−90.63 ± 6.89*) [−73.59 ± 11.83*]	(−78.26 ± 9.34*) [−71.69 ± 13.45*]	(−80.23 ± 10.08*) [−67.14 ± 12.34*]	(−95.26 ± 10.18*) [−87.54 ± 12.37*]	(−90.16 ± 8.95*) [−72.35 ± 11.89*]	(−89.26 ± 13.56*) [−72.12 ± 13.24*]

^a^Results are expressed as mean percentage ± S.E.M of three observations in each group; (% inhibition of AM); (% inhibition of FF).

Amylase inhibitory activity (%) was defined as the percentage decrease in maltose production rate over the control (without extract).

*Values significantly different (*P* < 0.05) from the control in each group without the extract added.

**Table 2:** Effects of AM and MC on *α*-glucosidase activity.

Concentration of extracts (*μ*g/mL)	*α*-Glucosidase activity % inhibition (−) or stimulation (+)^a^					
	Crude water Extract	Crude methanol extract	Dichloro-methane fraction	Ethyl acetate fraction	*n*-Butanol fraction	Water fraction
100	(−87.36±5.32)* [−68.96 ± 5.68*]	(−95.63 ± 6.23*) [−76.65 ± 2.36*]	(−94.63 ± 6.65*) [−89.59 ± 2.54*]	(−98.64 ± 7.42*) [−90.56 ± 8.54*]	(−72.63 ± 6.12*) [−87.65 ± 6.04*]	(−56.32 ± 2.36*) [−62.36 ± 6.54*]
50	(−76.32 ± 6.32*) [−54.36 ± 4.36*]	(−81.26 ± 7.12*) [−59.68 ± 5.67*]	(−74.12 ± 5.62*) [−76.63 ± 3.21*]	(−82.36 ± 2.34*) [−79.63 ± 7.54*]	(−52.32 ± 2.35*) [−62.38 ± 4.21*]	(−36.32 ± 3.36*) [−52.31 ± 2.35*]
25	(−52.36 ± 4.23*) [−36.12 ± 2.54*]	(−66.32 ± 2.36*) [−42.16 ± 2.15*]	(−69.32 ± 4.12*) [−60.15 ± 1.68*]	(−76.32 ± 6.32*) [−62.64 ± 6.21*]	(−40.16 ± 1.36*) [−40.13 ± 5.32*]	(−28.69 ± 1.12*) [−35.36 ± 3.57*]
12.5	(−32.15 ± 2.32*) [−28.69 ± 1.69*]	(−50.19 ± 1.13*) [−35.67 ± 1.69*]	(−52.14 ± 2.36*) [−40.19 ± 3.28*]	(−59.32 ± 5.32*) [−40.16 ± 3.54*]	(−36.94 ± 2.36*) [−27.68 ± 2.31*]	(−14.32 ± 1.62*) [−26.63 ± 2.07*]

^a^Results are expressed as mean percentage ± S.E.M of seven observations in each group; (% inhibition of AM); (% inhibition of MC).

The *α*-glucosidase inhibitory activity (%) was defined as the percentage decrease in absorbance over the control (without extract).

*Values significantly different (*P* < 0.05) from the control (without extract) in each group.

**Table 3:** Blood glucose level in glycogen-loaded mouse after oral administration of AM, FF, and MC extracts.

Samples	Without extract			Blood glucose concentration (mg/dl)^a^			
		Crude methanol extract		Crude water extract		Ethylacetate fraction	
		1 g/kg	2 g/kg	1 g/kg	2 g/kg	1 g/kg	2 g/kg
AM	375 ± 38	272 ± 19* [−27.5]	191 ± 14* [−49.1]	266 ± 21* [−29.1]	196 ± 13* [−47.8]	185 ± 17* [−50.1]	152 ± 19* [−59.4]
FF	386 ± 35	368 ± 37 [−4.7]	352 ± 32 [−8.8]	369 ± 35 [−4.4]	349 ± 22 [−9.6]	367 ± 39 [−4.9]	359 ± 29 [−7.0]
MC	379 ± 48	219 ± 29* [−42.2]	171 ± 39* [−54.9]	253 ± 26* [−33.2]	176 ± 13* [−53.6]	162 ± 11* [−57.3]	136 ± 15* [−64.1]

Acarbose	172 ± 19* [−55.1]		

^a^Results are expressed as means ± S.E.M of seven observations in each group. (% inhibition compared to respective control (without extracts added)).

*Values significantly different (*P* < 0.05) from the control in each group (before extract ingested). The average of blood glucose level without administration of extracts or glycogen was 157 ± 11 mg/dL. The dose of acarbose was 400 mg/kg.

**Table 4:** Phytochemicals components of AM, EM, FF, OT and PS leaves and MC fruits.

Plants	Alkaloids	Flavonoids	Tannins	Leucoanthocyanins	Anthraquinones	Terpenes	Phenols	Coumarins	Saponins
AM	+	+	+	+	−	−	+	−	+
EM	+	−	+	+	−	−	+	−	−
FF	−	+	+	−	−	+	−	−	−
PS	−	+	+	−	−	−	−	+	−
MC	+	+	+	+	−	−	+	−	+
OT	+	+	+	+	−	+	+	−	+

+ Presence, − absence.
